# Maternal high-fat diet programs offspring airway hyperinnervation and hyperresponsiveness

**DOI:** 10.1172/jci.insight.181070

**Published:** 2025-01-09

**Authors:** Kayla R. Williams, Hoyt A.T.K. Bright, Allison D. Fryer, David B. Jacoby, Zhenying Nie

**Affiliations:** Division of Pulmonary, Allergy, and Critical Care Medicine, Oregon Health & Science University, Portland, Oregon, USA.

**Keywords:** Metabolism, Pulmonology, Asthma, Innervation, Insulin

## Abstract

The impact of diet-induced maternal obesity on offspring airway hyperresponsiveness was studied in a diversity outbred mouse model that mirrors human genetic diversity. Female mice were started on high-fat or regular diet 8 weeks before breeding and throughout pregnancy and lactation. After weaning, all offspring were fed a regular diet. By 12 weeks, body weight and fat were increased in offspring of high-fat diet–fed dams, which was accompanied by metabolic dysfunction and hyperinsulinemia. This was followed by increased epithelial sensory innervation and increased bronchoconstriction to inhaled 5-hydroxytryptamine at 16 weeks. Bronchoconstriction was nerve mediated and blocked by vagotomy or atropine. A high-fat diet before pregnancy exerted the most influence on offspring airway physiology. Maternal obesity induced metabolic dysfunction and hyperinsulinemia, resulting in hyperinnervation and subsequent increased reflex-mediated hyperresponsiveness in their offspring. This is relevant to our understanding of asthma inheritance, considering the genetic diversity of humans.

## Abstract 2

Maternal obesity causes metabolic issues in offspring, including excess insulin levels, leading to heightened sensory innervation and airway sensitivity, possibly influencing asthma inheritance.

## Graphical abstract

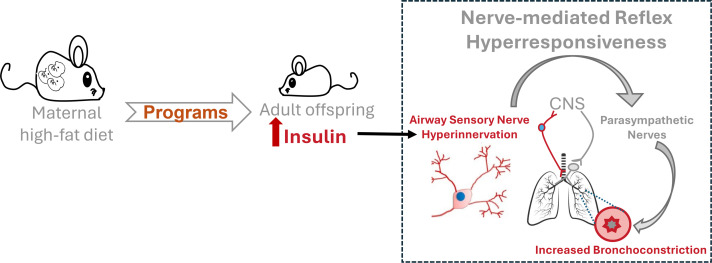

## Introduction

Children born to obese mothers in the United States are highly predisposed to developing asthma and obesity (1–6). Thirty-one percent of women in the United States are obese prior to pregnancy (7). Factors such as high maternal body mass index (BMI), increased maternal adiposity, high gestational weight gain, and the presence of type 2 diabetes or gestational diabetes can all predispose children to develop asthma, both during childhood and later in life (6, 8–11). But the exact mechanisms underlying asthma development in these offspring are not understood. Additionally, children born to mothers with obesity are more likely to have impaired metabolic function and obesity compared with children born to lean mothers (3, 5, 12, 13). This paper identifies a mechanism that explains how the transgenerational burden of maternal obesity affects asthma development in their adult offspring.

Asthma characteristics include airway hyperresponsiveness (14, 15), which is the result of hyperfunctional airway nerves that control bronchoconstriction (airway narrowing). Bronchoconstriction has been shown to be more pronounced in asthma and is mediated through a reflex that includes both airway sensory and parasympathetic nerves (16). Airway sensory nerves are distributed throughout the airway epithelium and send signals to the central nervous system to activate parasympathetic nerves, which release the neurotransmitter acetylcholine onto M_3_ muscarinic receptors on airway smooth muscle. This then induces smooth muscle contraction resulting in bronchoconstriction. Parasympathetic nerves are also abnormal in asthma, with loss of inhibitory M_2_ muscarinic receptors on the nerves (15, 17–19). Nerve-mediated bronchoconstriction is increased in all animal models of asthma, including exposure to ozone (20, 21) or organophosphate pesticides (22), viral infection (23, 24), antigen challenge (25), and obesity (26, 27)

Obesity is a major risk factor for asthma (28, 29). Obesity-associated insulin resistance has been linked to increased airway reactivity (27), reduced lung function (30), and poor asthma control (28, 29, 31, 32). Our previous studies in animal models of diet-induced obesity demonstrate that insulin drives increased airway reactivity (27, 33, 34) and that selective depletion of insulin receptors on airway sensory nerves prevents both airway sensory hyperinnervation and hyperresponsiveness (26). Obesity is more common in children born to obese mothers (13), along with features of metabolic syndrome (35, 36), hyperglycemia (36), insulin resistance (32), and hyperinsulinemia (37). Previous animal studies (38–40) have shown that maternal high-fat diet or high-calorie diet exposure is linked with persistent development of metabolic dysfunction and respiratory complications in offspring. Although all these studies investigated the impact of an adverse intrauterine environment on the metabolic health of offspring, they provide limited insight into the mechanisms driving development of respiratory complications in offspring born to obese mothers. In particular, the neural contributions to airway hyperresponsiveness remain insufficiently explored.

Here, we used a diversity outbred mouse model, which mimics human genetic diversity, combined with diet-induced maternal obesity, to pinpoint the onset of metabolic dysfunction in relation to changes in airway nerve morphology and responsiveness in offspring. This study aims to validate and expand upon our previous observational findings from an inbred mouse strain (40), which demonstrated airway hyperresponsiveness in offspring of obese dams. By verifying the causal relationship between metabolic dysfunction and airway responsiveness in offspring, this study provides an in-depth mechanistic investigation, offering insights into how maternal obesity programs airway hyperresponsiveness in adult offspring.

## Results

### Maternal HFD increases dams’ body fat.

**Figure 1:**
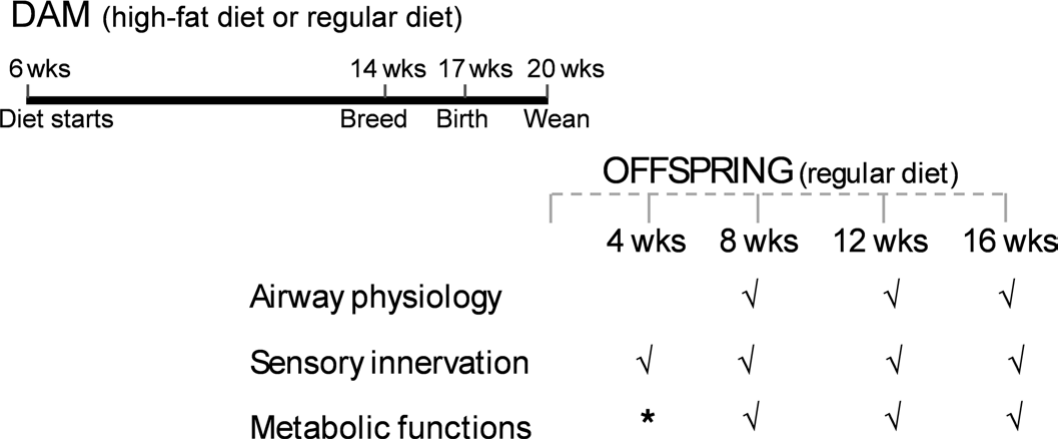
Measuring airway hyperresponsiveness in offspring of obese dams. Female mice were put on a high-fat diet (HFD) from 6 to 20 weeks of age, including during breeding, pregnancy, and lactation (black line). Offspring were weaned at 3 weeks and switched to a regular diet (RD) until 16 weeks (gray line). Offspring body weight, body fat, fasting insulin, and epithelial sensory innervation were measured at 4, 8, 12, and 16 weeks. Offspring airway physiology, fasting glucose, glucose tolerance, and insulin tolerance were measured at 8, 12, and 16 weeks. *Represents partial data collected for the respective time point.

**Figure 2:**
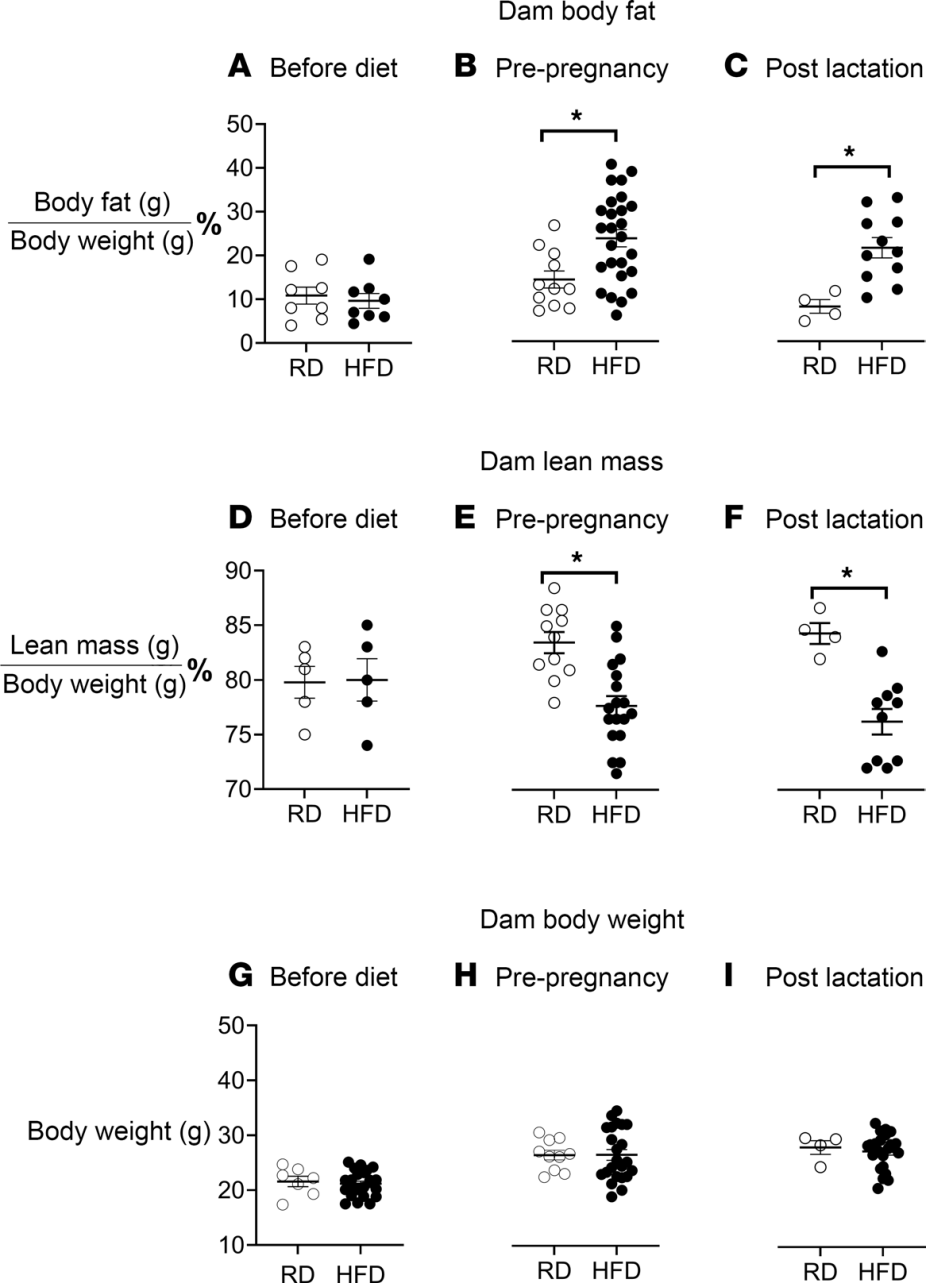
Dams on an HFD develop increased body fat. Before dietary intervention, all dams exhibited similar body fat (**A**), body lean mass (**D**), and body weight (**G**). Dams on an HFD (filled circles) had significantly increased body fat versus dams on an RD (unfilled circles) from before pregnancy (**B**) through the end of lactation (**C**). In contrast, body lean mass was significantly reduced in dams on an HFD compared with dams on an RD from before pregnancy (**E**) through the end of lactation (**F**). However, the HFD did not result in a significant increase in body weight compared to dams on an RD throughout the entire duration of the diet treatment (**G**–**I**). Each circle represents 1 animal, with *n* = 4–11 for the RD and *n* = 8–26 for the HFD. Data were analyzed by Student’s *t* test and presented as means ± SEM. *P < 0.05.

At 6 weeks of age, before any dietary intervention (Figure 1), mice showed no significant difference in body fat (Figure 2A), lean mass (Figure 2D), or body weight (Figure 2G). Female mice fed an HFD (filled circles) gained significantly more body fat than female mice fed an RD (unfilled circles), as measured at 14 (after 8 weeks of diet and before breeding, Figure 2B) and 20 weeks of age (after lactation, Figure 2C). In contrast, lean mass was significantly decreased in female mice fed an HFD compared with female mice fed an RD at 14 and 20 weeks of age (Figure 2, E and F). Body weight increased with age (between 6 and 20 weeks) for all females, but there were no significant diet-related differences in body weight (Figure 2, H and I).

Fasting blood glucose was measured in a subpopulation of dams at the end of lactation. Dams fed an HFD had a significant increase in fasting glucose levels compared with dams fed an RD (145.0 ± 7.859 mg/dL in HFD dams, *n* = 11, vs. 109.8 ± 7.859 mg/dL in RD dams, *n* = 11; *P* < 0.05). There was no difference in glucose tolerance tests among females, irrespective of their diet.

Dams were mated with unrelated male mice, resulting in each female mouse delivering a single litter. Among dams on an RD, litter sizes ranged 7–12 pups/litter (mean = 9.5 ± 0.6 SEM), while dams on an HFD produced litters with 3–10 pups (mean = 7.1 ± 0.6 SEM, Table 1). We used more dams on an HFD because of their smaller litter size.

### Hyperinsulinemia in offspring of HFD-fed dams.

**Figure 3:**
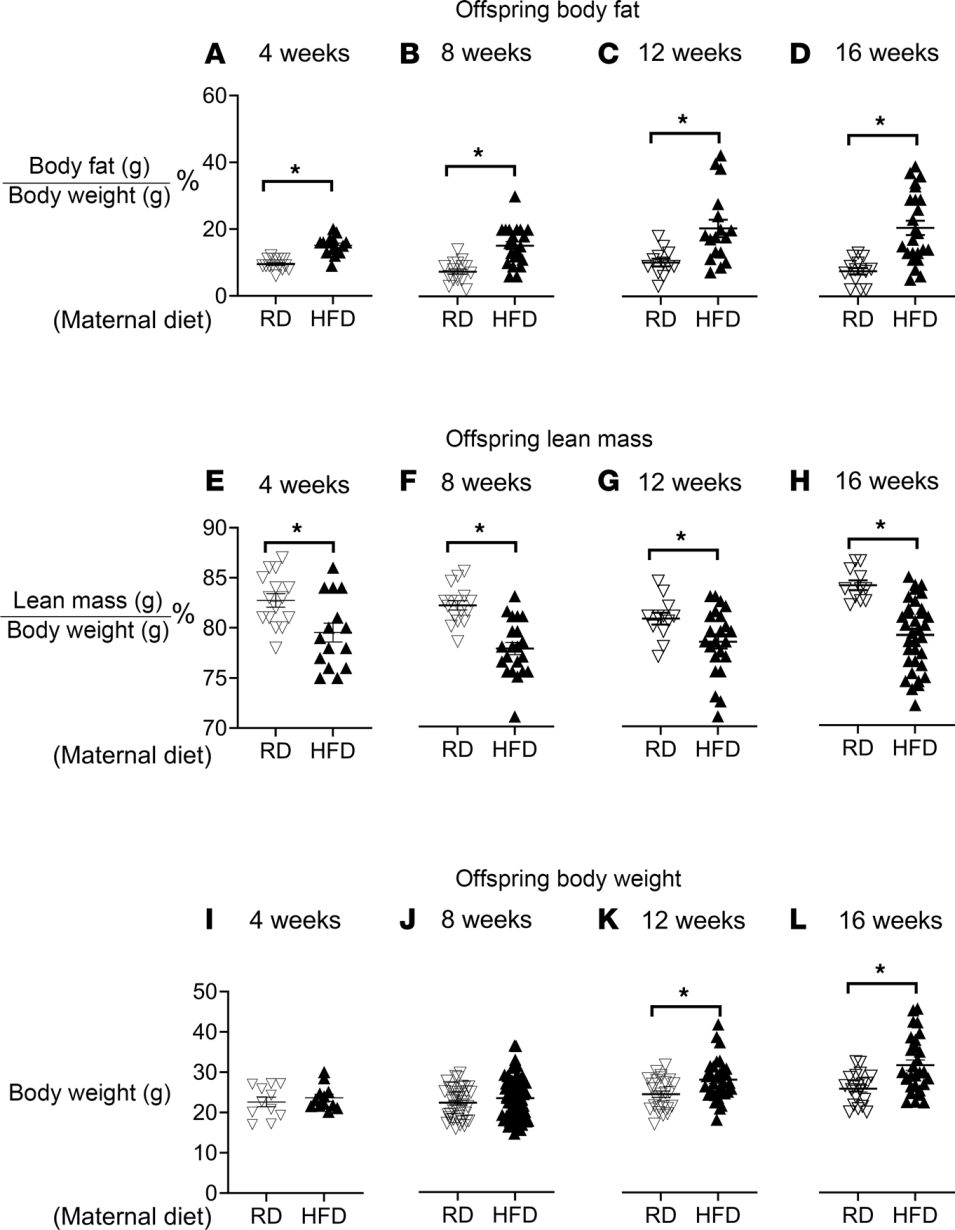
Offspring born to dams on an HFD develop obesity. Offspring of dams on an HFD (filled triangles) had significantly increased percentage body fat (**A**–**D**) and decreased percentage lean mass (**E**–**H**) at all time points measured (ages 4–16 weeks) compared with offspring of dams on an RD (unfilled triangles). There was no significant difference in body weight (**I** and **J**) until 12 weeks, after which offspring of dams on an HFD increased their weight, and this persisted through 16 weeks (**K** and **L**). Each triangle represents 1 animal; *n* = 10–28 for offspring of dams on an RD, and *n* = 11–39 for offspring of dams on an HFD. Data were analyzed by Student’s *t* test and presented as means ± SEM. *P < 0.05.

Body fat, expressed as a percentage of body weight, exhibited a consistent and significant increase in offspring from dams on an HFD at all time points (filled triangles, Figure 3, A–D). Conversely, lean mass, expressed as a percentage of body weight, in offspring of HFD dams showed a significant decrease from 4 to 16 weeks of age compared with offspring of dams fed an RD (Figure 3, E–H). Although offspring born to dams on an HFD weighed significantly less than offspring born to dams on a regular-fat diet (mean 1.403 g ± 0.06676 g SEM in offspring of HFD dams versus mean 1.547 g ± 0.06676 g SEM in offspring of RD dams, Table 1), they caught up to their peers by 4 weeks of age (Figure 3, I and J) and eventually surpassed them by 12 weeks (Figure 3, K and L).

**Figure 4:**
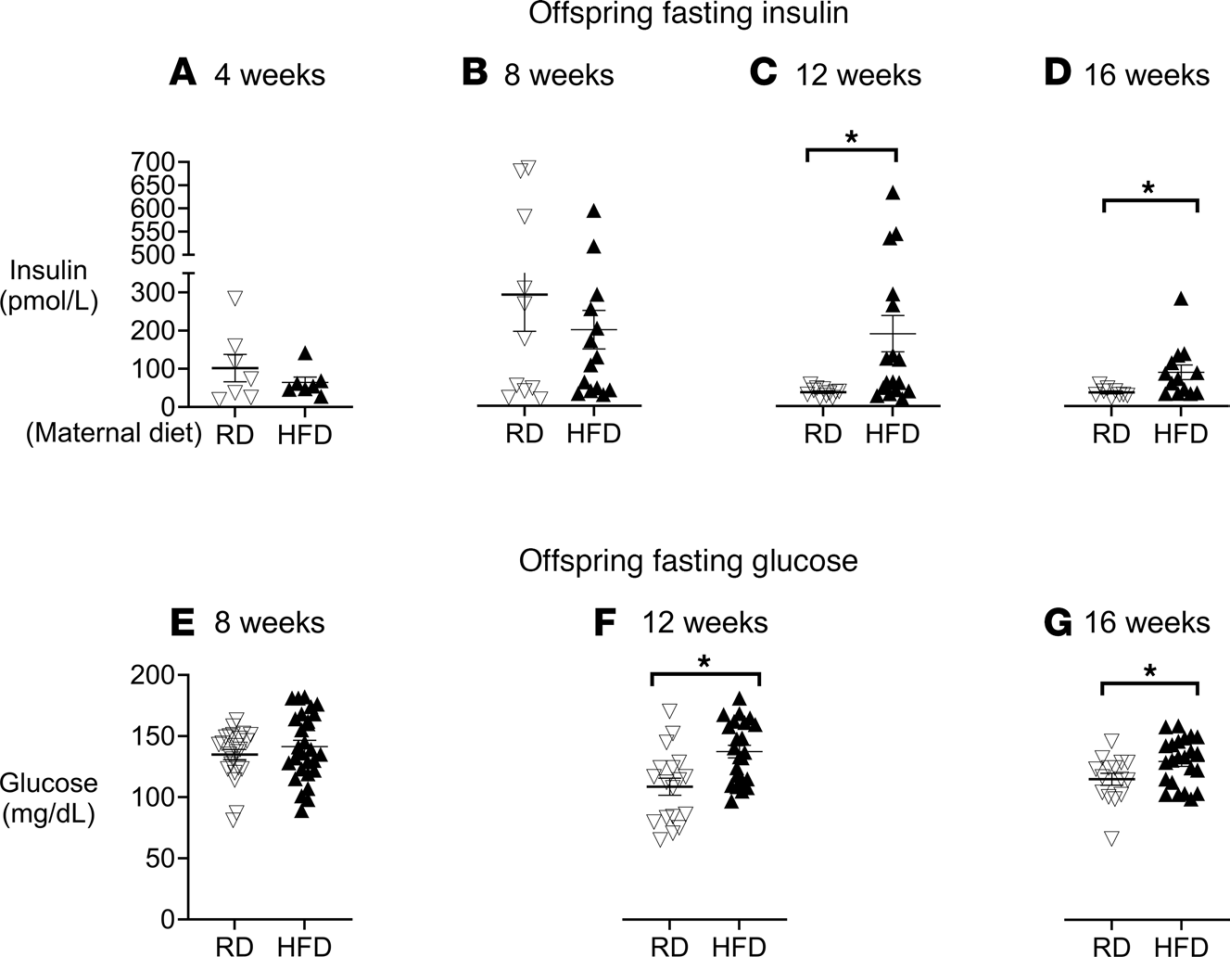
Hyperglycemia and hyperinsulinemia in offspring of HFD dams. Offspring groups show no difference in fasting circulating insulin between 4 and 8 weeks of age (**A** and **B**). Offspring of HFD dams (filled triangles) have significantly higher fasting circulating insulin at 12 and 16 weeks of age compared with offspring from dams on an RD (unfilled upside-down triangles) (**C** and **D**). Offspring of dams on an HFD (filled triangles) do not have elevated fasting glucose at 8 weeks of age compared to offspring from dams on an RD (unfilled upside-down triangles) (**E**) but develop elevated glucose at 12 and 16 weeks of age (**F** and **G**). Each triangle represents 1 animal; *n* = 6–24 for offspring of dams on an RD, and *n* = 7–35 for offspring of dams on an HFD. Data were analyzed by Student’s *t* test and presented as means ± SEM. *P < 0.05.

All offspring showed similar fasting insulin at 4 weeks of age (Figure 4A), and offspring from dams on an RD did not exhibit any change with age (4–16 weeks, Figure 4, A–D). In contrast, offspring of dams on an HFD (Figure 4) had a significant increase in fasting insulin compared with those born from dams on an RD at 12 and 16 weeks of age (Figure 4, C and D).

Similarly, fasting glucose in offspring of dams on an RD (Figure 4) did not differ significantly over time (4–16 weeks) (Figure 4, E–G). Fasting glucose was similar between groups at 8 weeks but was significantly increased in offspring of dams on an HFD (Figure 4E) compared with an RD (Figure 4, F and G) at 12 and 16 weeks of age.

**Figure 5:**
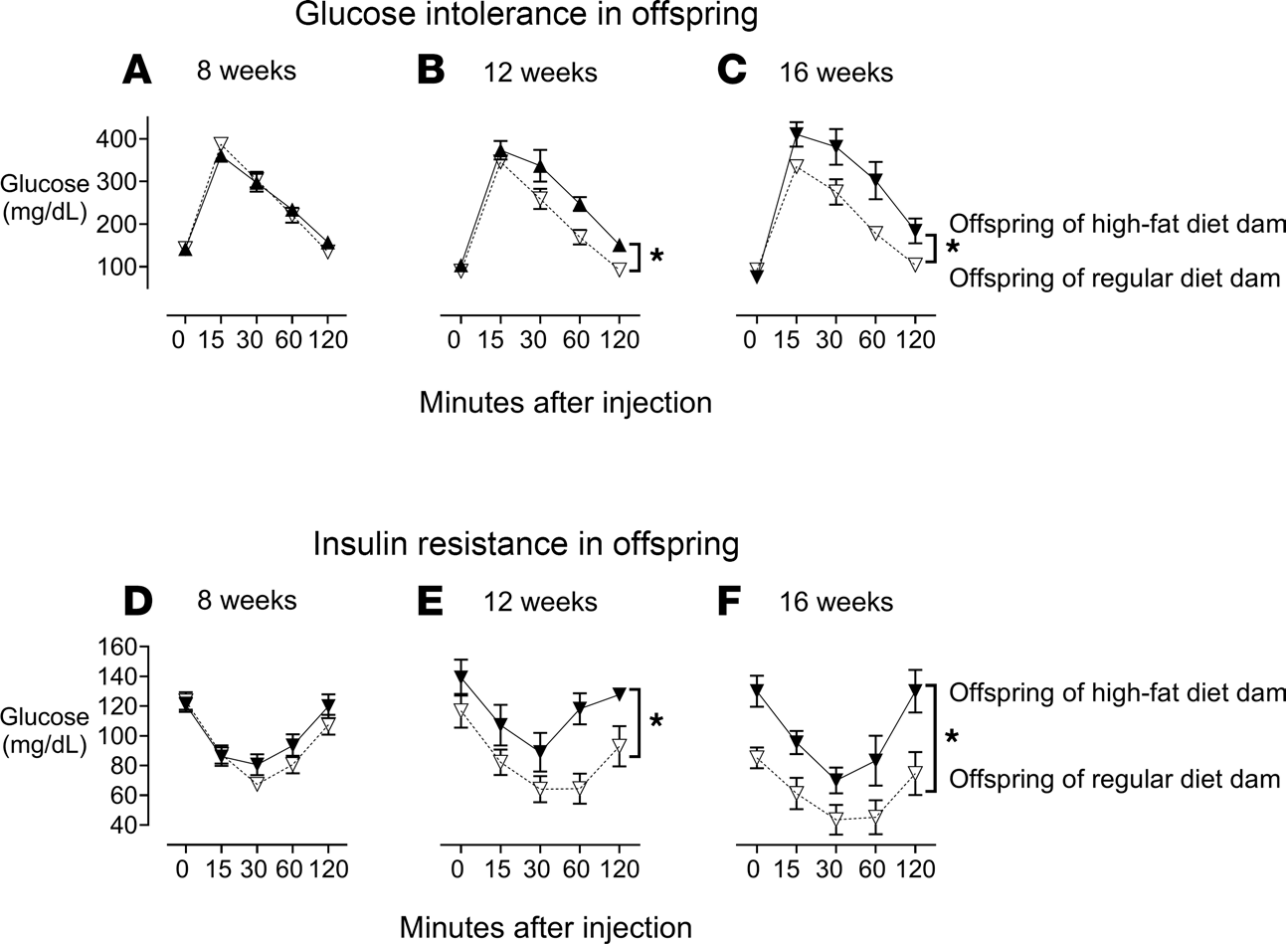
Impaired glucose tolerance and insulin resistance in offspring of HFD dams. Offspring of HFD dams (filled triangles) have significantly impaired responses to glucose and insulin starting at 12 weeks of age compared with offspring from dams on an RD (unfilled upside-down triangles) (**B**, **C**, **E**, and **F**). There were no differences in glucose tolerance or insulin tolerance at 8 weeks of age (**A** and **D**). Each data point represents the mean ± SEM; *n* = 5–14; *P < 0.05, using a 2-way ANOVA.

Glucose tolerance and insulin tolerance were tested in offspring at 8, 12, and 16 weeks of age (Figure 5). Offspring of dams on an RD had normal glucose tolerance and insulin tolerance throughout the ages 4 to 16 weeks (Figure 5, A–F). Glucose tolerance and insulin tolerance at 8 weeks of age were similar between offspring of dams on an HFD and offspring of dams on an RD (Figure 5, A and D). However, by 12 and 16 weeks, offspring of dams on an HFD had significantly impaired responses to both glucose (Figure 5, B and C) and insulin (Figure 5, E and F) compared with offspring of dams on an RD.

### Increased airway reflex bronchoconstriction in offspring from HFD-fed dams.

**Figure 6:**
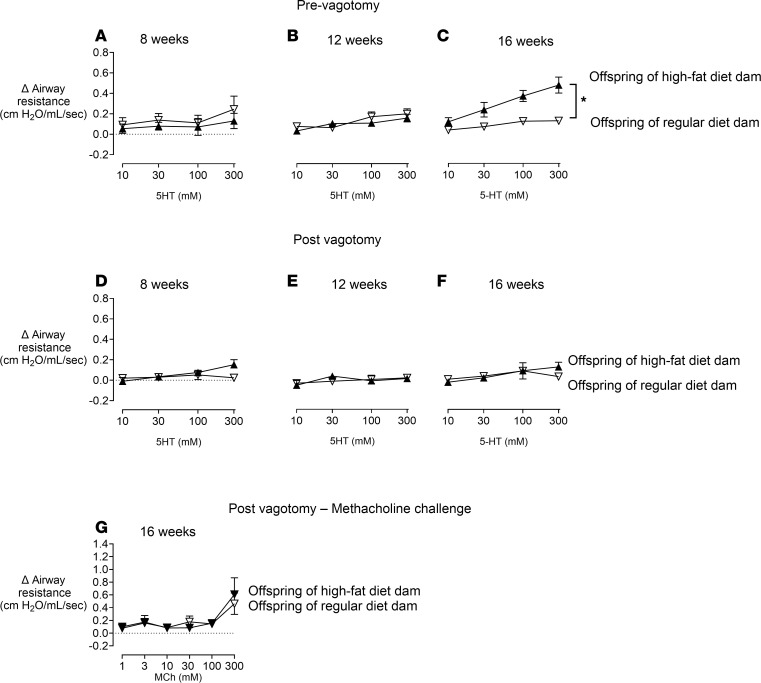
Airway hyperresponsiveness in offspring of HFD dams. Bronchoconstriction induced by inhaled 5-hydroxytryptamine (5-HT) was not different between groups at 8 or 12 weeks (**A** and **B**) but was significantly increased at 16 weeks in offspring of dams fed an HFD (**C**, black triangles). This was blocked by vagotomy (**D**–**F**), indicating that airway nerves mediate 5-HT–induced bronchoconstriction. Bronchoconstriction induced by methacholine (MCh) was not significantly different between groups at 16 weeks of age (**G**). Each data point represents the mean ± SEM; *n* = 5–14; *P < 0.05, using a repeated 2-way ANOVA.

Airway responsiveness to airway stimuli was measured at 8, 12, and 16 weeks of age (Figure 6). In offspring of dams on an RD, inhalation of aerosolized 5-HT (10–300 mM) induced a small, dose-dependent bronchoconstriction, measured as an increase in airway resistance, which remained consistent across groups of different age (Figure 6, A–C). Bronchoconstriction induced by aerosolized 5-HT was not significantly increased in offspring of dams on an HFD at 8 (Figure 6A) or 12 weeks (Figure 6B) of age compared to those of dams on an RD. However, by 16 weeks of age, offspring of dams on an HFD had significantly increased bronchoconstriction in response to aerosolized 5-HT (Figure 6C). Vagotomy significantly inhibited 5-HT–induced bronchoconstriction (Figure 6, D–F), demonstrating that 5-HT triggers reflex bronchoconstriction mediated by the vagus nerves. After vagotomy, inhaled MCh-induced bronchoconstriction was similar between offspring born from dams on an HFD versus dams on an RD, indicating no changes of M_3_ muscarinic receptor function on airway smooth muscle (Figure 6G). Bronchoconstriction was also blocked by atropine, demonstrating that release of acetylcholine from parasympathetic nerves plays a key role in airway hyperresponsiveness in offspring of dams on an HFD at 16 weeks of age (Supplemental Figure 2; supplemental material available online with this article; https://doi.org/10.1172/jci.insight.181070DS1).

### Increased airway epithelial sensory innervation in offspring from HFD-fed dams.

**Figure 7:**
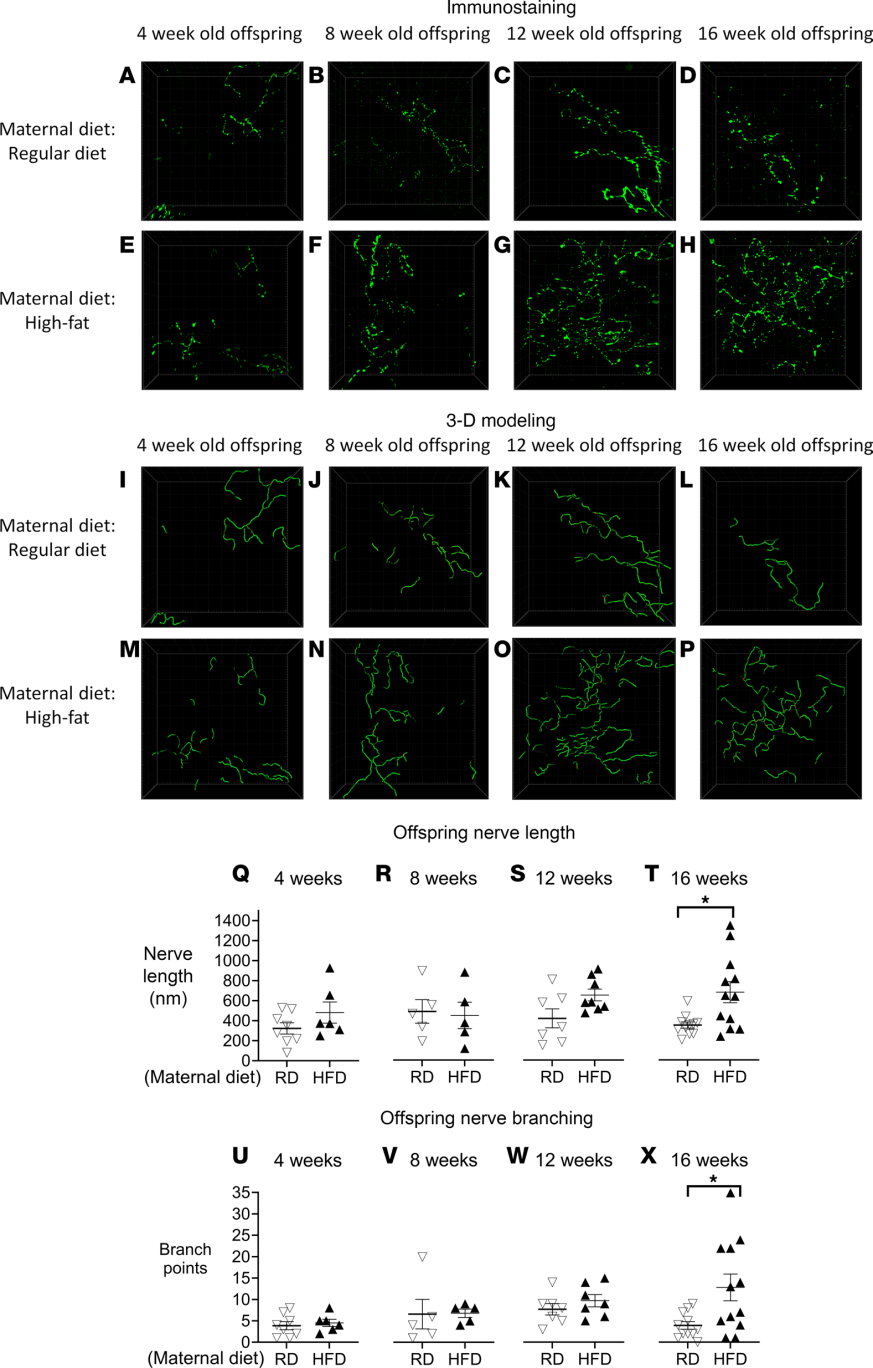
Offspring of dams on an HFD (filled triangles) have increased airway epithelial sensory innervation at 16 weeks old when compared with offspring of dams on an RD (unfilled upside-down triangles). Whole trachea from offspring of dams fed an HFD or RD were stained using antibodies against PGP 9.5 (green), optically cleared, and imaged using confocal microscopy. Representative images are shown (**A**–**H**). Imaris modeling software was used to create 3D models of airway nerves in **A**–**H**, shown in **I**–**P**, respectively. Sensory innervation was quantified by measuring nerve length (**Q**–**T**) and nerve branching (**U**–**X**). By 16 weeks, offspring from dams on an HFD had significantly increased nerve length and branching (**T** and **X**). Each triangle represents 1 animal; *n* = 7–11 for offspring from dams on the RD, and *n* = 5–12 for offspring from dams on the HFD. Data were analyzed by Student’s *t* test and presented as means ± SEM. *P < 0.05.

Based on expression of the pan-neuronal marker PGP 9.5 (Figure 7, A–H), 3-dimensional (3D) models of airway epithelial nerves were created, and nerve branch points were identified by Imaris modeling software (Figure 7, I–P). In offspring of dams on an RD (Figure 7, Q–X), there was no change in sensory nerve length or branching with increasing age (4–16 weeks). Epithelial sensory nerve length and branching were also similar between offspring of dams on HFD and offspring of dams on RD at 4 and 8 weeks of age (Figure 7, Q, R, U, and V). However, offspring of dams on an HFD (Figure 7, C and D) displayed a trend toward increases in both nerve length and branching starting at 12 weeks of age (filled triangles, Figure 7, S and W) that culminated in a significant increase in both nerve length and branching by 16 weeks of age compared with that in offspring of dams on an RD (Figure 7, T and X).

### Neuronal M_2_ muscarinic receptor protein expression was similar among all offspring.

**Figure 8:**
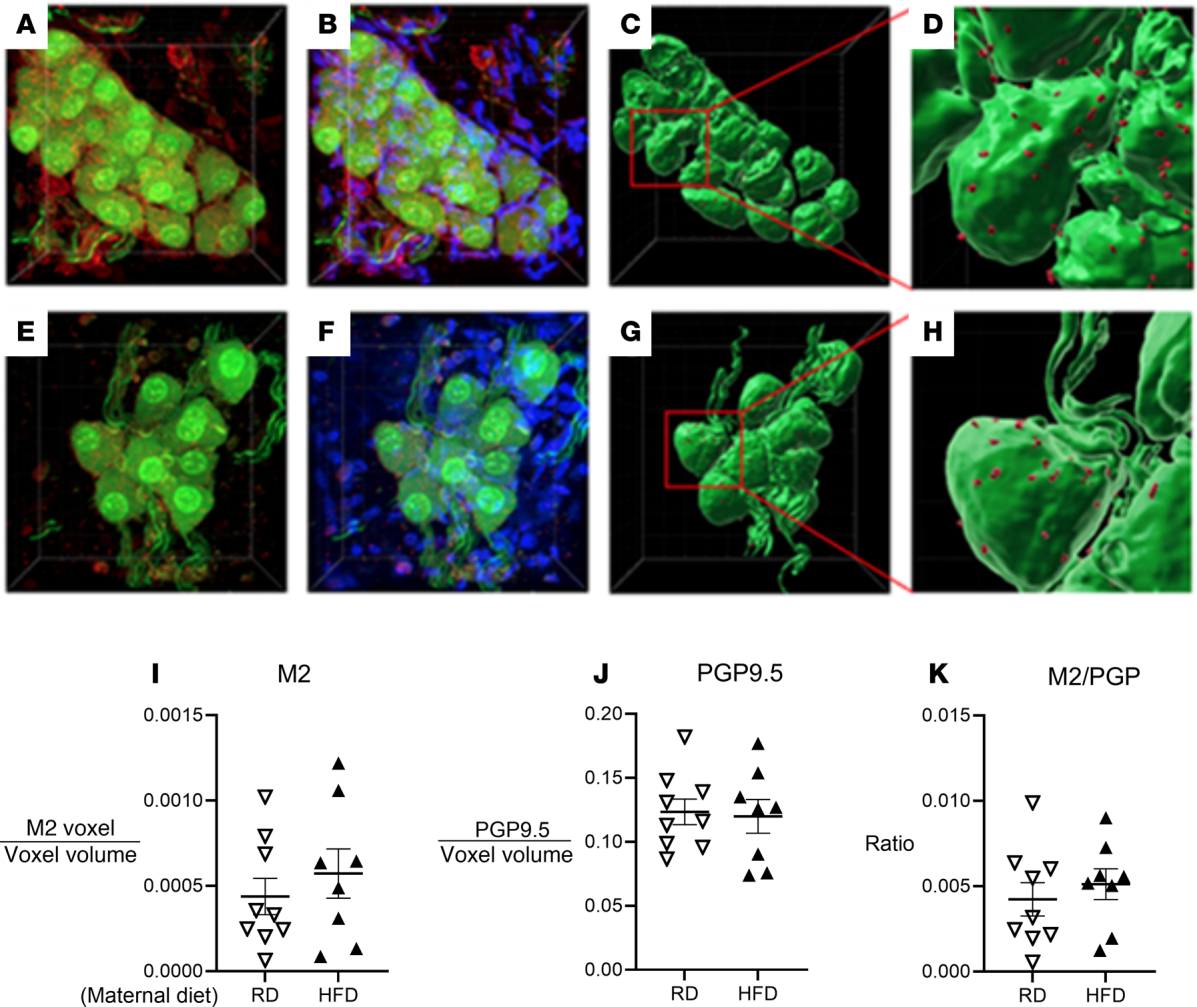
Offspring of dams on an HFD (filled triangles) have similar neuronal M_2_ muscarinic receptor protein expression at 16 weeks old when compared to offspring of dams on an RD (unfilled upside-down triangles). Neuronal ganglia in trachea isolated from offspring mice born to dams on an RD (**A**–**D**) and an HFD (**E**–**H**) were identified by antibody against the pan-neuronal marker PGP 9.5 (green). M_2_ muscarinic receptors were identified by antibody against RFP (red). Nuclei were stained with DAPI (**B** and **F**). Images were computationally modeled into 3D representations of neuronal ganglia clusters (**C**, **D**, **G**, and **H**). The neuronal M_2_ muscarinic receptors are shown on the cell membranes (**D** and **H**). The total expression of neuronal M_2_ muscarinic receptor protein (voxels) on airway ganglia (**I**) is comparable between offspring born from dams on an HFD and those on an RD. There was no difference in volume of ganglia (voxel) of airway ganglia (**J**) between offspring born from dams on an HFD and RD. Normalized neuronal M_2_ muscarinic receptor expression of PGP 9.5–stained nerves did not show significant differences among offspring (**K**). Data were analyzed by Student’s *t* test and presented as means ± SEM. *P < 0.05.

Neuronal M_2_ muscarinic receptor protein expression, identified by the coexpression of red fluorescent protein (RFP) and the pan-neuronal marker PGP 9.5, was visualized on the cell membrane (Figure 8). These receptors were found on or across the cell membrane of parasympathetic nerve ganglia and nerve fibers but not inside the cell (Figure 8, D and H). There was no significant difference in neuronal M_2_ muscarinic receptor protein expression (Figure 8I) and PGP 9.5 expression (Figure 8G) between offspring born from dams on an HFD and those from dams on an RD. To account for variations in the number and size of ganglion cells, M_2_ muscarinic receptor expression in airway parasympathetic ganglia was normalized to PGP 9.5 expression (Figure 8K). There was no significant difference in neuronal M_2_ receptor expression on airway parasympathetic neurons between the 2 groups.

### Effects of maternal exposure to an HFD during critical windows of offspring development: pre-pregnancy, pregnancy, and post-pregnancy (lactation) periods.

**Figure 9:**
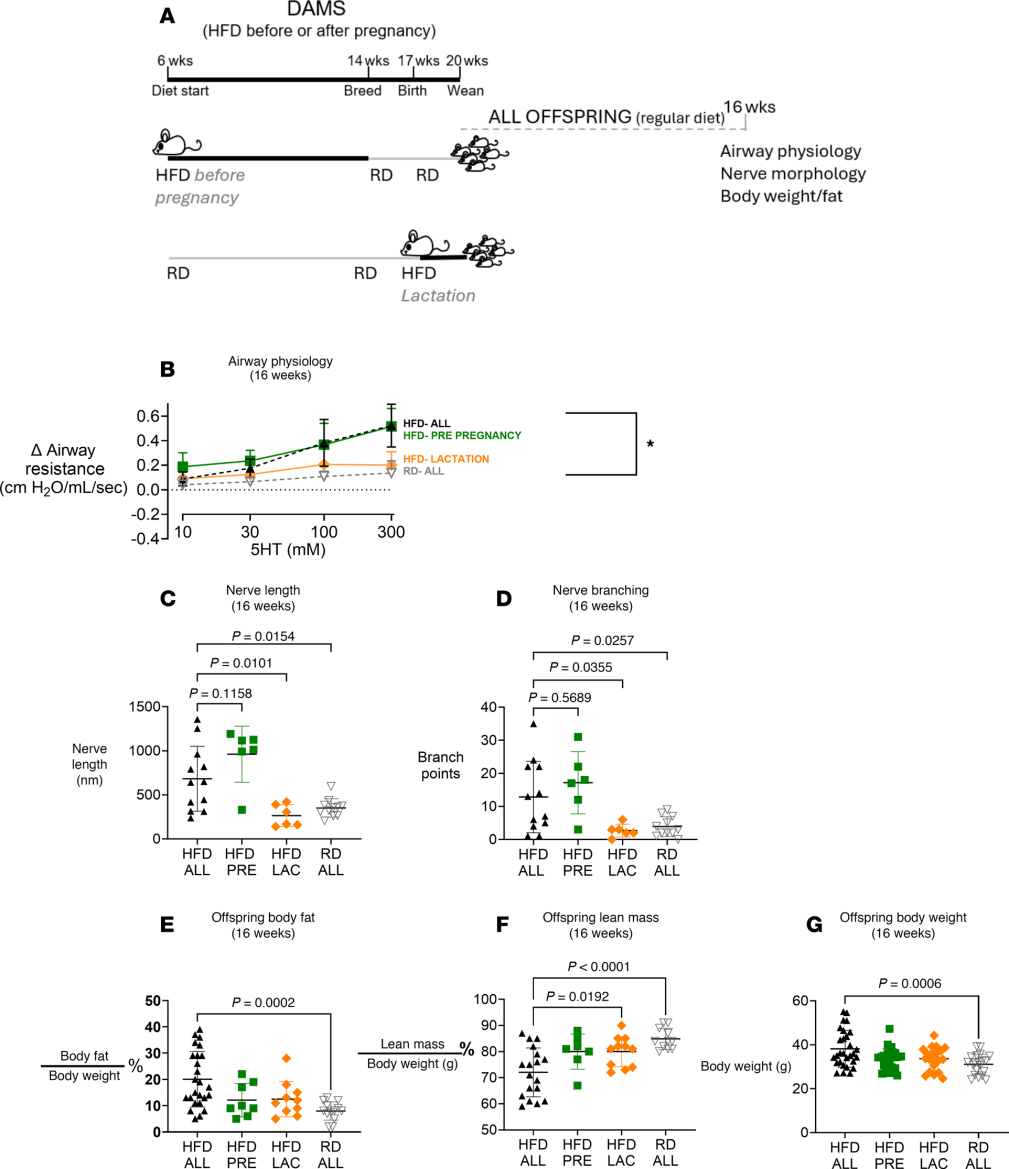
Measuring airway hyperresponsiveness in offspring of dams given an HFD only during specific times critical for offspring development. (**A**). Female mice were fed an HFD before breeding only, during lactation only, or throughout pre-pregnancy, pregnancy, and lactation. Female mice fed an HFD or RD from 8 weeks before breeding until the end of lactation served as the controls. Offspring of mice fed an HFD before breeding only have similar increased airway response to 5-HT to offspring of mice fed HFD before, during, and after pregnancy (the latter data are reproduced from Figure 5 for ease of comparison). Bronchoconstriction in response to inhaled 5-HT in 16-week-old offspring born from dams on an HFD only during lactation was similar to that in offspring born from dams on an RD (**B**). Airway epithelial sensory nerve density was increased in offspring of dams fed an HFD before pregnancy, as well as those fed an HFD throughout pre-pregnancy, pregnancy, and lactation, but not in those fed an HFD during lactation only (**C** and **D**). In offspring of dams fed an HFD during all periods, body fat (**E**) was increased, lean mass (**F**) was decreased, and body weight (**G**) was increased. None of these changed in offspring of dams fed an HFD only pre-pregnancy or during lactation. Offspring physiology data were analyzed using a repeated 2-way ANOVA. Each data point represents the mean ± SEM, with *n* = 5–14; *P < 0.05. For offspring metabolic parameters, each symbol represents 1 animal, with *n* = 5–28. Data were analyzed using a 1-way ANOVA and presented as means ± SEM.

To identify the critical windows of susceptibility to maternal dietary influences on offspring outcomes, dams were exposed to an HFD during specific periods — pre-pregnancy (8 weeks before pregnancy) and during lactation. Subsequently, metabolic parameters and reflex bronchoconstriction were measured in offspring at 16 weeks of age, allowing a comparison with offspring born to dams on a consistently high- or regular-fat diet (data from offspring in Figure 6C were reproduced in this experiment to facilitate comparison) (Figure 9).

The offspring of dams exposed to an HFD only during lactation had airway responsiveness similar to the offspring of dams on an RD (Figure 9B). However, offspring of dams on an HFD before pregnancy only were similar to offspring of dams on the HFD during all periods (pre-pregnancy and pregnancy and lactation). These offspring developed airway hyperresponsiveness, and there were no differences in responsiveness among these groups (Figure 9B). Subsequent vagus nerve transection still demonstrated substantial inhibition of 5-HT–induced bronchoconstriction in all offspring, verifying the role of airway nerves in (reflex) bronchoconstriction induced by 5-HT (data not shown). These offspring also developed increased airway epithelial sensory nerve density quantified as increased nerve length (Figure 9C) and number of branch points (Figure 9D) compared with offspring of lean dams but were not significantly different from offspring from obese dams.

Offspring from dams on an RD before pregnancy showed trends of reduced body fat (Figure 9E), increased lean mass (Figure 9F), and lower body weight (Figure 9G) compared with those born to dams on an HFD during pre-pregnancy and lactation. However, these trends were not statistically significant. In contrast, offspring exposed to an HFD only during lactation exhibited significantly higher body lean mass (Figure 9F) and no significant differences in body fat (Figure 9E) or weight (Figure 9G) compared to those exposed throughout pre-pregnancy and lactation.

## Discussion

We have established a model of diet-induced maternal obesity using genetically diversity outbred mice, which mimic the genetic diversity of human populations. Offspring of dams on an HFD, even when the offspring were exposed solely to an RD, exhibited a gradual increase in visceral adiposity, body weight, fasting glucose, and fasting insulin. This was followed by increased airway epithelial sensory innervation and airway hyperresponsiveness. Our current data show that the impact of maternal obesity on offspring airway hyperresponsiveness is consistent across genetically diverse backgrounds. Additionally, we showed that airway hyperresponsiveness in offspring of dams on an HFD is not present in early life but emerges later following increased adiposity and hyperinsulinemia. This supports the causal relationship between metabolic dysfunction and airway responsiveness in offspring. Furthermore, our data show that exposure to an HFD, whether limited to pre-pregnancy or extended into pregnancy, leads to metabolic changes and airway hyperresponsiveness in offspring. The results of our study contribute to a deep understanding of how asthma characteristics develop in offspring born to obese individuals and lay the foundations for developing effective prevention and treatment strategies for this population.

The increased airway hyperresponsiveness observed in offspring of dams on an HFD is mediated by airway nerves because bronchoconstriction triggered by inhaled 5-HT exhibited a substantial increase in these offspring compared with those on an RD when vagus nerves were intact. Vagotomy abolished this heightened bronchoconstriction, verifying the involvement of vagus nerves. Sensory nerve density in the airway epithelium was increased in offspring of dams on an HFD (Figure 7). As increased airway sensory nerve density is associated with airway hyperresponsiveness both in animal models and in humans with asthma (16, 40, 41), it is likely the increased bronchoconstriction induced by aerosolized 5-HT in offspring of HFD dams stems from the marked increase in sensory nerve density in airway epithelium.

The similar bronchoconstriction induced by inhaled MCh across vagotomized offspring, regardless of maternal diet, indicates the airway hyperresponsiveness in offspring of dams on an HFD was not mediated by changes in M_3_ muscarinic receptor function on airway smooth muscle. We also showed that all offspring had similar airway expression of neuronal M_2_ muscarinic receptors (Figure 8), which is critical for modulating parasympathetic nerve function.

The observed airway nerve–mediated hyperresponsiveness in offspring of dams on an HFD is likely induced by hyperinsulinemia. High circulating insulin, acting through insulin receptors on sensory nerves, promotes growth and increased density of airway sensory nerves in airway epithelium (40). High insulin levels may also affect airway efferent parasympathetic nerves, as in previous studies showing that increased insulin correlates with airway hyperreactivity in rats with diet-induced obesity (27, 42). Those studies also showed a contribution of increased function of parasympathetic nerves. Release of acetylcholine onto excitatory M_3_ muscarinic receptors on airway smooth muscle is normally limited by inhibitory M_2_ muscarinic receptors on parasympathetic nerves (43). Loss of M_2_ receptor function is associated with increased acetylcholine release and increased bronchoconstriction, which has been shown in obese rats with hyperinsulinemia (27). Decreasing insulin in obese rats by treating with streptozotocin restores neuronal M_2_ muscarinic receptor function (27). Thus, high levels of insulin cause airway hyperresponsiveness via affecting both sensory and parasympathetic nerves. Our current study reveals that hyperinsulinemia precedes emergence of increased airway epithelial sensory innervation and, subsequently, airway hyperresponsiveness, reinforcing the causal link between hyperinsulinemia and airway hyperresponsiveness. Additionally, our data show that offspring of dams on an HFD during pre-pregnancy or throughout all pregnancy phases exhibited increased airway reflex bronchoconstriction to inhaled 5-HT, even though their body fat and body weight did not significantly differ from control offspring, validating our previous finding that airway hyperreactivity is mediated by a mechanism independent of fat accumulation (27).

Human studies have established a link between maternal obesity or being overweight and increased risk of asthma in offspring (44–47). Epidemiological data show the association between maternal obesity and increased risk of wheezing and asthma from newborn to early adulthood (46, 48, 49). However, there is also an association of maternal obesity as an indicator of both early and late onset of obesity in these children, including increased risk of metabolic dysfunction, insulin resistance, and type 2 diabetes. Upon closer scrutiny, it is apparent the pivotal factor is not gestational weight gain but rather maternal body fat. Harpsøe et al. reported in a cohort study involving 38,874 mother-child pairs from the Danish National Birth Cohort that maternal BMI, rather than gestational weight gain, was associated with an elevated risk of asthma and wheezing in offspring (44). Similarly, in another population-based longitudinal cohort study, Polinski et al. reported the same conclusion (46). Building on these human studies, our investigation delved into the impact of maternal HFD of dams concerning offspring airway hyperresponsiveness. Our data reveal that dams exposed to an HFD, whose offspring exhibited airway hyperresponsiveness, displayed a noticeable increase in maternal body fat, while their body weight remained statistically not different from control dams on an RD. This finding reinforces our argument that maternal BMI emerges as a more critical determinant than gestational weight gain for predicting asthma risk in offspring. Our study underscores the significance of focusing on maternal BMI as a key factor in understanding intergenerational implications of dietary influences on respiratory health.

Our data reveal that intrauterine exposure to maternal obesity triggers a distinct, progressive pattern of physiological changes after birth, culminating in neurally mediated airway hyperreactivity in adult offspring of obese dams. The impact of maternal obesity on offspring respiratory health is intricately tied to timing of exposure. Neither neuronal development nor lung function was affected by exposure to an HFD only during lactation. In contrast, offspring of mothers on an HFD only pre-pregnancy (which may have a continued effect during gestation) developed airway hyperinnervation and airway hyperresponsiveness similar to airway hyperreactivity in human cohorts (50, 51). Our data provide a mechanism explaining the implications of diet and obesity in pregnancy on intergenerational respiratory health of their offspring.

Maternal obesity continues to rise across the world (6, 44, 45, 47). The escalating rates of asthma in children, currently 14% worldwide, may be linked to these increases in maternal obesity (6, 44, 45, 47). We have shown increased sensory nerve density in the lung of eosinophilic asthma in human populations (52). Here we show that sensory nerve hyperinnervation is also a mechanism for asthma in offspring of mothers on an HFD. However, rather than triggering neuronal growth via eosinophils, maternal obesity more likely initiates this response via in utero exposure to insulin. We propose that strategies aimed at reducing maternal obesity, decreasing fat in maternal diets during pregnancy, or preventing hyperinsulinemia may help prevent development of asthma in their offspring.

## Methods

### Sex as a biological variable.

Both male and female mice were used in this study. In accordance with the consideration of sex as a biological variable, data were further analyzed by sex, with results presented in Supplemental Figures 1–4.

### Animals.

Diversity outbred mice (strain 009376) (purchased from Jackson Laboratory) and Chrm2-tdT-D mice that express an M_2_ muscarinic receptor/tdTomato fusion protein directed by the endogenous M_2_ muscarinic receptor promoter/enhancer sequences (gift from Hongkui Zeng, Allen Institute for Brain Science, Seattle, Washington, USA) were housed in a pathogen-free facility on a 12-hour light/12-hour dark cycle with free access to food and water. To model maternal obesity, 6-week-old females were fed either an RD (13.6% kcal from fat, LabDiet PicoLab Laboratory Rodent Diet 5L0D) or an HFD (45% kcal from fat, Inotiv catalog TD.06415). After 8 weeks of dietary intervention, females were bred with unrelated males fed with an RD. Maternal diets were continued during breeding, pregnancy, and lactation, until pups were 3 weeks old and weaned (Figure 1).

To identify which critical windows of maternal HFD affect offspring lung function, some 6-week-old female diversity outbred mice were randomly assigned to the HFD for pre-pregnancy only (8 weeks) or for lactation only (3 weeks) (Figure 9) and were given an RD during other breeding phases. Upon weaning, all offspring were fed an RD, and physiological measurements were taken at 16 weeks only (Figure 9; only 1 time point was used for these animals). Data from these animals were compared with the data from 16-week-old offspring of dams fed an HFD or RD for the entire pre-pregnancy, pregnancy, and lactation periods. For the majority of the project, offspring of dams fed an HFD or RD throughout all pregnancy phases were used to measure differences in the metabolic, morphologic, and physiologic measurements detailed below.

For all experiments, offspring from the same litter were randomly assigned to different experimental procedures (as in Figure 1). Some offspring were used for physiological measurements of airway function, as well as measuring fasting glucose and glucose tolerance. Their littermates were used to measure sensory innervation in the trachea, as well as measuring fasting insulin and insulin tolerance. All offspring were fasted for 16 hours before physiological measurements were taken, including metabolic measurements, tissue harvesting, and airway physiology.

### Metabolic measurements.

Body weights were recorded before and after dietary interventions in dams (6–20 weeks of age) and offspring (4–16 weeks of age). Body fat and lean mass were measured by nuclear magnetic resonance (Echo MRI) as previously described (33). Fasting glucose was measured using a handheld glucose meter (OneTouch Ultra2, LifeScan, Inc.). Fasting plasma insulin was measured by ELISA (mouse insulin ELISA, Lot 35395, Mercodia), using serum collected from the abdominal vein of anesthetized animals.

Glucose tolerance tests were performed by measuring blood glucose before and 5, 15, 20, 30, 60, 90, and 120 minutes after administering 2 mg/kg of 20% glucose prepared in phosphate-buffered saline (PBS) solution (i.p.). Insulin tolerance tests were performed by measuring blood glucose at the same time points after administering 0.075 U/kg of 0.075 U/mL insulin in PBS solution (i.p.).

### Measuring reflex-mediated bronchoconstriction in vivo.

Mice were anesthetized using ketamine (100 mg/kg i.p.) and xylazine (10 mg/kg i.p.). Mice were tracheotomized, paralyzed using succinylcholine (10 mg/kg i.p.), and mechanically ventilated with 100% oxygen, constant flow, 0.2 mL tidal volume, and 120 breaths per minute with a positive end-expiratory pressure of 2 cmH_2_0. Body temperature was monitored using a rectal thermometer and maintained at 36°C–37°C using a heating lamp and heating pad. Heart rate was monitored with an electrocardiogram. Inspiratory flow was measured via pneumotachograph (MLT1L, ADInstruments), and airway pressure was recorded with a pressure transducer (MLT0670, ADInstruments). Data were recorded using a PowerLab 4/SP analog-to-digital converter and analyzed with the LabChart Pro software (ADInstruments). Airway resistance was measured as previously described (40). Two deep inhalations were given between each dose of aerosolized saline (via a nebulizer) or 5-HT (10 μL, 10–300 mM, MilliporeSigma, via a nebulizer). Following each deep inhalation, a series of inspiratory pauses at peak inspiration were given for 225 ms to measure plateau pressures in the airways. For each breath, airway resistance in cmH_2_O/mL/s was calculated using the formula (P_peak_ – P_plateau_)/inspiratory flow. Six breaths were averaged before and 50 seconds after administration of either aerosolized saline or 5-HT. In the same animal, measurement of airway responses to aerosolized 5-HT was repeated after cutting both vagus nerves, or 5 minutes after treating with atropine (3 mg/kg, i.p.). Reflex contributions to bronchoconstriction were measured by comparing 5-HT–induced increases in airway resistance before and after vagotomy, as previously described (26, 40). Additionally, the role of acetylcholine was verified by blocking with atropine.

### Measuring M_3_ muscarinic receptor–induced bronchoconstriction in vivo.

Mice were anesthetized, ventilated, and paralyzed, and the vagus nerves on both sides of the trachea were cut. Airway resistance was measured before and after inhaled MCh (10 μL, 1–300 mM, MilliporeSigma, via a nebulizer), with after-MCh measurement taken once peak pressure reached a plateau after each dose of inhaled MCh. M_3_ muscarinic receptor activation–induced bronchoconstriction was calculated by subtracting the baseline airway resistance from the airway resistance measured after each dose of MCh.

### Immunostaining, tissue optical clearing, imaging, and measuring airway epithelial sensory nerves and M_2_ muscarinic receptors on airway ganglia.

Airway epithelial sensory innervation was detected using immunostaining and analyzed by Imaris modeling software as previously described (40, 53). Offspring were anesthetized with ketamine (100 mg/kg i.p.) and xylazine (10 mg/kg i.p.), then perfused through the right ventricle with PBS (10 mL). Trachea and bronchi were harvested and fixed with Zamboni fixative (Newcomer Supply) overnight at 4°C. Whole tracheas were opened along the center of the ventral side, and some of the excess cartilage was trimmed off to allow the trachealis to lie flat. Tissues were washed 5 times for 1 hour with 1× TBS, followed by overnight or 3-day blocking with 4% normal goat serum, 1% Triton X-100, and 0.05% powdered milk. Subsequently, the tracheas were incubated with primary antibodies (PGP 9.5, rabbit IgG, 1:250, Abcam [epithelial nerves] catalog ab108986; rabbit anti-PGP 9.5 [neuronal ganglia], 1:250 dilution, MilliporeSigma catalog AB1761-1; chicken anti-RFP, 1:500 dilution, Novus Biologicals, Bio-Techne, NP1-97371) in blocking solution for 1 to 3 days on a shaker at 4°C. After 5 additional 1-hour washes, the tracheas were incubated overnight with secondary antibodies (Alexa Fluor goat anti-rabbit 488, 1:1,000; catalog number A-11008, Invitrogen, Thermo Fisher Scientific; or Alexa Fluor goat anti-chicken 647, 1:1,000; catalog number A32933, Invitrogen, Thermo Fisher Scientific) in blocking solution in the dark. Tracheas were then counterstained using the nuclear stain DAPI (dilactate; Invitrogen, Thermo Fisher Scientific) at room temperature for 10 minutes in the dark. Tracheas were then optically cleared in N-methylacetamide/Histodenz (Ce3D) overnight, rendering them transparent, and whole mounted in Ce3D on slides in 120 μm–deep imaging wells (catalog number S24735, Invitrogen, Thermo Fisher Scientific).

Airway nerves were identified by PGP 9.5 staining, and nuclei of airway epithelial cells were identified by DAPI staining. Confocal *z*-stack images were taken at the bifurcation of each offspring trachea with a ZEISS LSM900 confocal microscope (with a ×63/1.4 oil PlanApo DIC M27 objective with a 0.19 nm working distance) using the center *z*-stack function. Using Imaris modeling software (Imaris 9.9.1, Oxford Instruments), 2 to 3 *z*-stack images of the airway epithelial layer with the same dimensions and 3 *z*-stack images of neuronal ganglia were used to create 3D models of airway nerves using the manual filament function. Nerve length and branch points were quantified by Imaris, and the value of 2 images taken from same trachea were averaged to quantify sensory nerve density in the trachea of each animal. Three images per mouse trachea were quantified, and average values from these images were considered representative data for each animal.

In mice that express M_2_ muscarinic receptors fused to a tdTomato protein, M_2_ muscarinic receptors were recognized by RFP staining. Neuronal M_2_ muscarinic receptor expression was quantified by generating a surface around M_2_ muscarinic receptor and PGP 9.5 colocalized voxels, measuring the volume of voxels in contact with PGP 9.5–positive neuron models. Total neuronal M_2_ receptor expression in 3D images was quantified in voxel units. To normalize neuronal M_2_ receptor expression to neuron number, the ratio of M_2_ muscarinic receptor voxels to PGP 9.5 voxels was calculated.

### Statistics.

As there was no sex difference between body fat or body weight (Supplemental Figure 1), these data were combined in Figures 1–8 above. All data were analyzed using Prism 10 software (GraphPad). Airway resistance in response to inhaled 5-HT or MCh, glucose tolerance, and insulin tolerance were analyzed using a 2-way ANOVA with a repeated measurement and Bonferroni’s post hoc test. Fasting glucose, fasting serum insulin, body weight, body fat percentage, lean mass, nerve length, nerve branching, and the M_2_ muscarinic receptor expression were analyzed using unpaired 2-tailed Student’s *t* test. *P* values less than 0.05 were considered significantly different. The results were expressed as mean ± SEM.

### Study approval.

Animals were handled in accordance with protocols approved by the Institutional Animal Care and Use Committee at Oregon Health & Science University.

### Data availability.

The data supporting the findings of this study are available in the Supporting Data Values file. Additional information, including the analytic code, can be provided upon request.

## Author contributions

ZN conceived the study. ZN and KRW designed the study. KRW, HATKB, and ZN conducted the experiments. KRW and ZN analyzed the data and wrote the manuscript. ADF and DBJ assisted in directing the study, contributed to data analysis, and edited the manuscript. All authors gave their approval for the final manuscript.

## Supplementary Material

Supplemental data

Supporting data values

## Figures and Tables

**Table 1:**
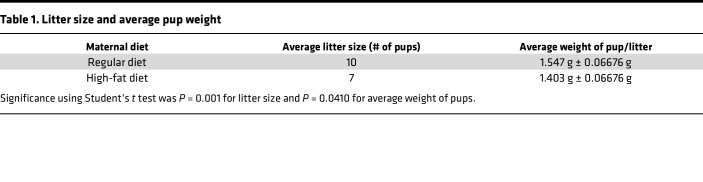
Litter size and average pup weight

## Version 1 01/09/2025

Electronic publication
