# Supplementary material for: Maternal high-fat diet programs offspring airway hyperinnervation and hyperresponsiveness
Source: JCI Insight. 2025 Jan 9;10(1):e181070. doi: 10.1172/jci.insight.181070 (PMC11721309; doi:10.1172/jci.insight.181070)
Supplement: Supplemental data [file jciinsight-10-181070-s077.pdf]

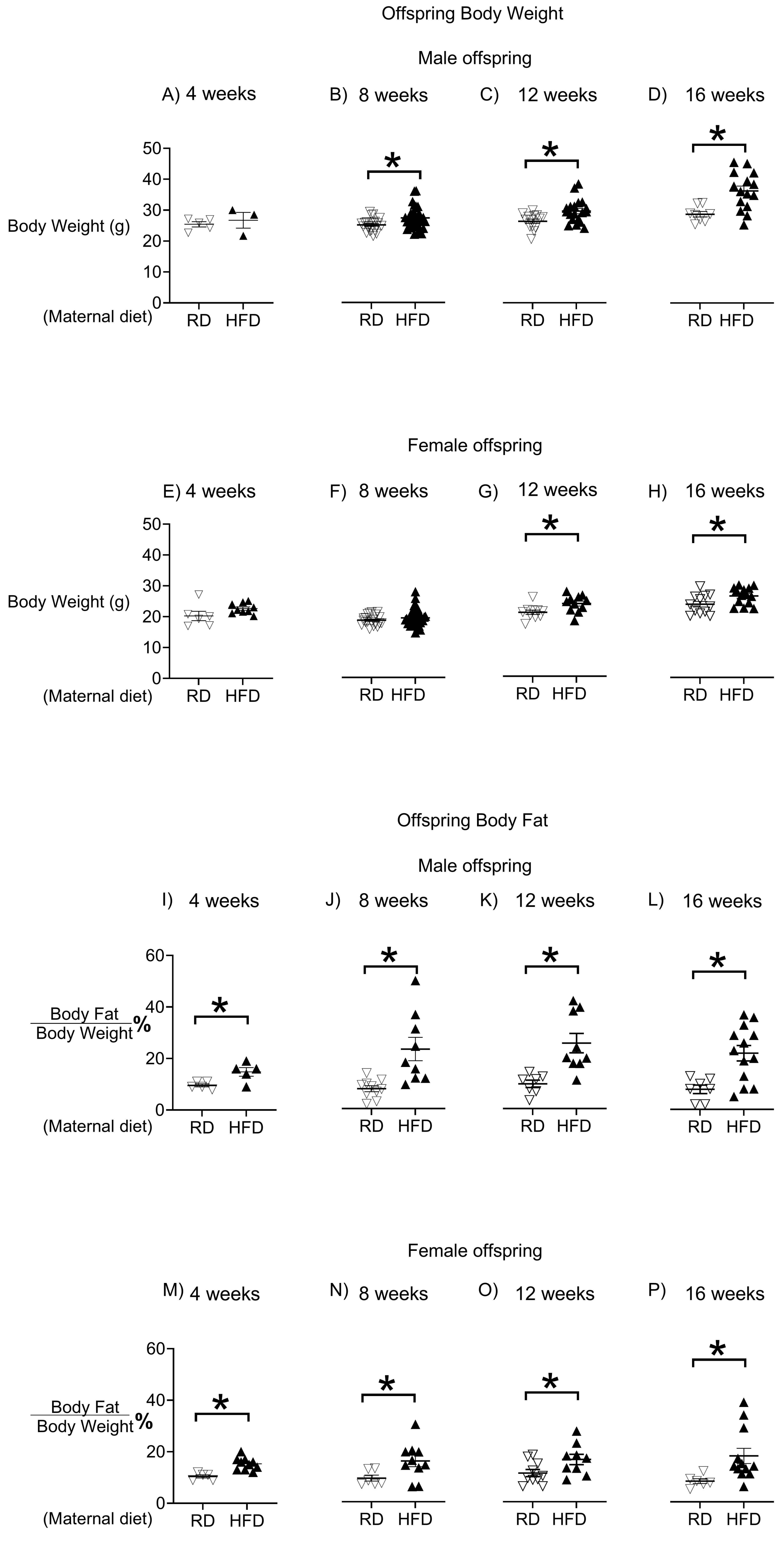

Offspring Body Weight
Male offspring
A) 4 weeks
B) 8 weeks
C) 12 weeks
D) 16 weeks
Body Weight (g)
(Maternal diet)
RD
HFD
Female offspring
E) 4 weeks
F) 8 weeks
G) 12 weeks
H) 16 weeks
Offspring Body Fat
Male offspring
I) 4 weeks
J) 8 weeks
K) 12 weeks
L) 16 weeks
Body Fat / Body Weight %
Female offspring
M) 4 weeks
N) 8 weeks
O) 12 weeks
P) 16 weeks

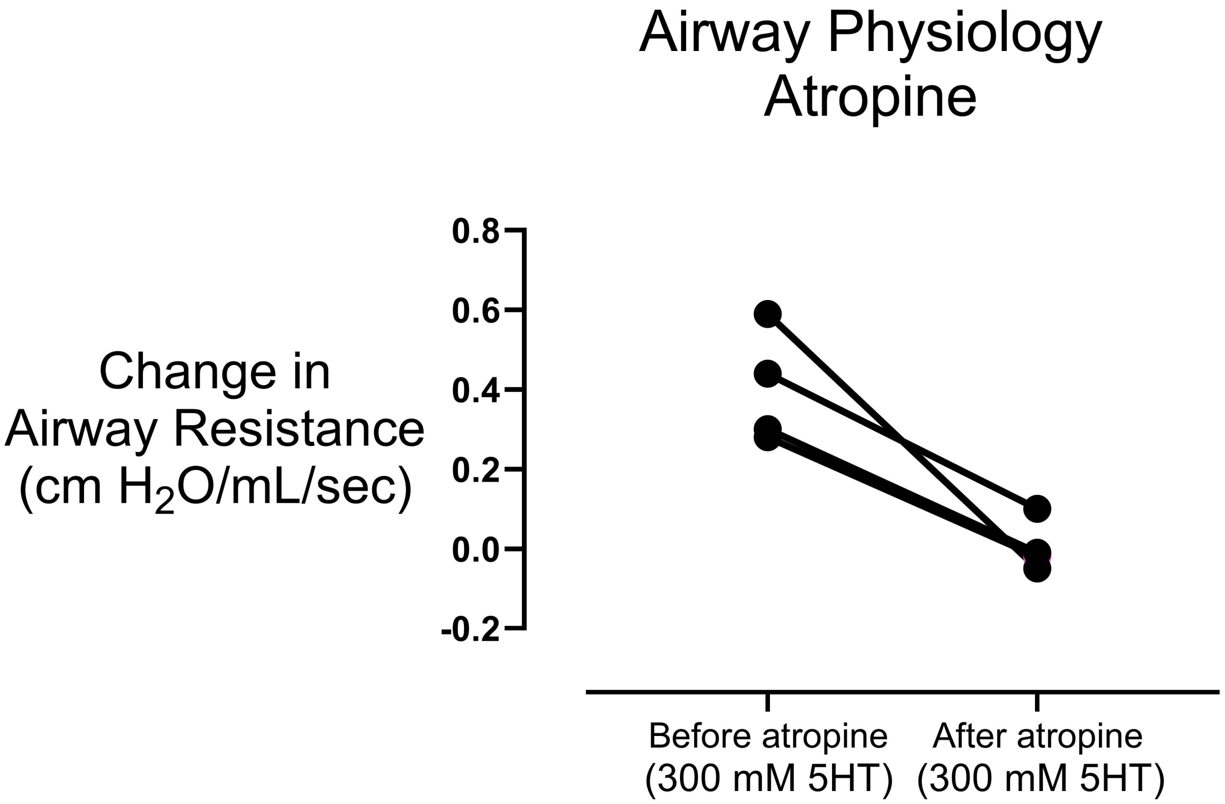

Airway Physiology
Atropine
Change in
Airway Resistance
(cm H2O/mL/sec)
0.8
0.6
0.4
0.2
0.0
-0.2
Before atropine
(300 mM 5HT)
After atropine
(300 mM 5HT)

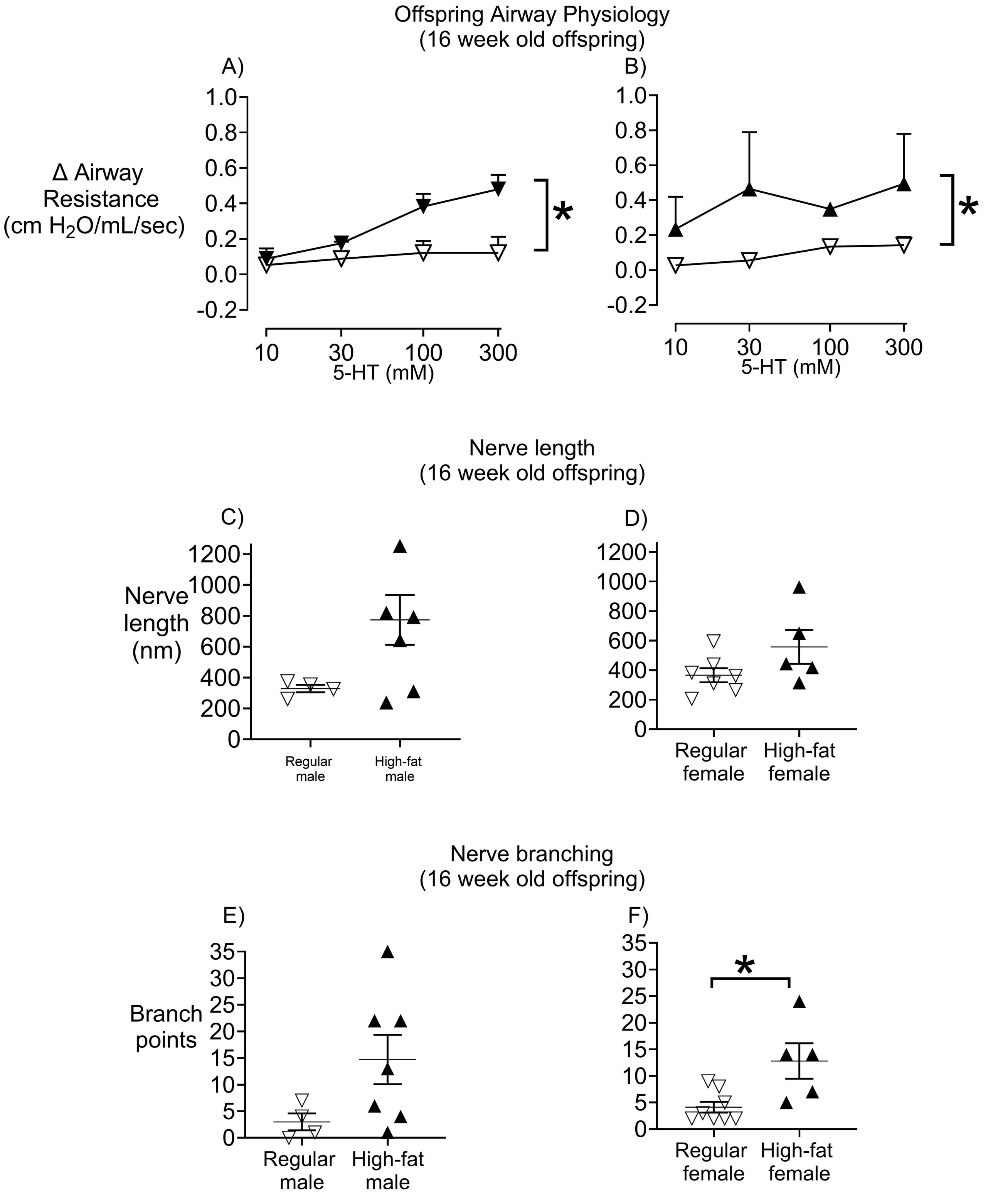

Offspring Airway Physiology
(16 week old offspring)
A)
B)
Δ Airway Resistance (cm H2O/mL/sec)
1.0
0.8
0.6
0.4
0.2
0.0
-0.2
10
30
100
300
5-HT (mM)
*
Nerve length
(16 week old offspring)
C)
D)
Nerve length (nm)
1200
1000
800
600
400
200
0
Regular male
High-fat male
Regular female
High-fat female
Nerve branching
(16 week old offspring)
E)
F)
Branch points
35
30
25
20
15
10
5
0
Regular male
High-fat male
Regular female
High-fat female
*

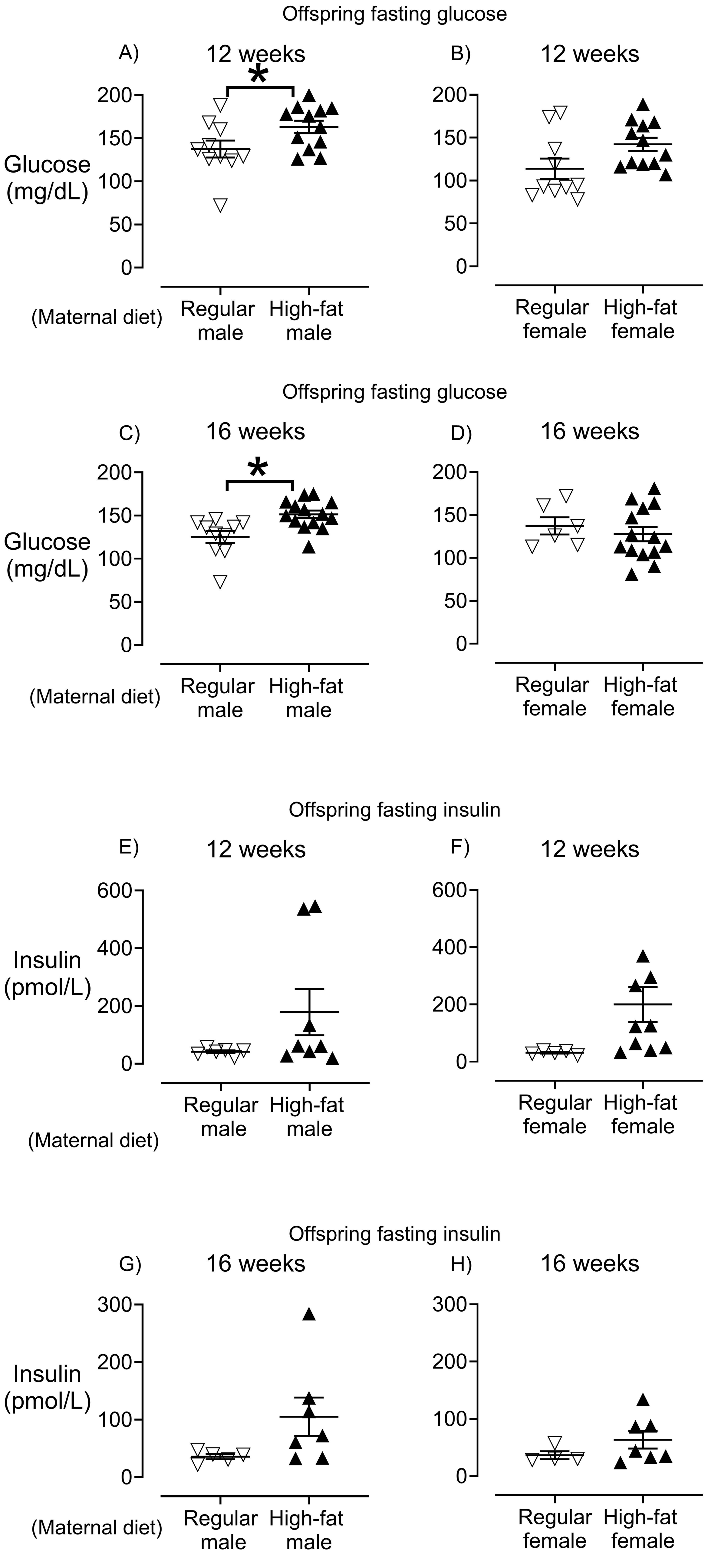

Offspring fasting glucose
A) 12 weeks
B) 12 weeks
Glucose (mg/dL)
200
150
100
50
0
*
(Maternal diet)
Regular male
High-fat male
Regular female
High-fat female
Offspring fasting glucose
C) 16 weeks
D) 16 weeks
Offspring fasting insulin
E) 12 weeks
F) 12 weeks
Insulin (pmol/L)
600
400
200
0
Offspring fasting insulin
G) 16 weeks
H) 16 weeks
300
100
